# The Impact of Serum 25‐Hydroxyvitamin D on Bone Mineral Density at Different Skeletal Sites: A Multivariate Analysis Based on a UK Cohort Study

**DOI:** 10.1002/fsn3.71894

**Published:** 2026-05-17

**Authors:** Hualian Zeng, Xinning Tong, Shuangnan Cui, Chen Shen, Jinhui Li, Qisheng Lin, Yuejiao Huang, Xiaoxin I. Yao

**Affiliations:** ^1^ Department of Orthopaedics The Eighth Affiliated Hospital, Sun Yat‐Sen University Shenzhen China; ^2^ Department of Epidemiology, School of Public Health Sun Yat‐Sen University Guangzhou China; ^3^ MRC Centre for Environment and Health, Department of Epidemiology and Biostatistics, School of Public Health Imperial College London London UK; ^4^ National Institute for Health Research Health Protection Research Unit in Radiation Threats and Hazards Imperial College London London UK; ^5^ School of Public Health, Division of Environmental and Occupational Health Sciences University of Illinois Chicago Chicago Illinois USA; ^6^ Department of Civil and Environmental Engineering The Hong Kong Polytechnic University Hong Kong SAR China

**Keywords:** bone mineral density, cohort study, multivariate analyses, vitamin D

## Abstract

Vitamin D is essential for bone health, but its effects on bone mineral density (BMD) may exhibit regional variations. This study aims to ascertain whether a consistent relationship exists between serum vitamin D and BMD at various bone sites. Participants were recruited from the UK Biobank, a large prospective cohort study. Serum 25‐hydroxyvitamin D levels were measured at baseline (2006–2010), and BMD at the lumbar, pelvis, spine, femur, and arm were evaluated via DXA. A multivariate regression analysis was employed to examine the relationship between serum 25‐hydroxyvitamin D and BMD at five sites simultaneously. This retrospective cohort study included 20,131 participants. The fully adjusted model revealed that a 1 nmol/L increase in serum 25‐hydroxyvitamin D was associated with a 0.24, 0.38, 0.37, 0.28, and 0.20 mg/cm^2^ higher BMD at the spine, lumbar, pelvis, femur, and arm, respectively. The multivariate regression demonstrated that serum vitamin D strongly correlated with the BMD at the lumbar and pelvis regions, followed by the femur, spine, and arm. The associations were significantly different by gender, with smaller impacts observed in females compared to males at the pelvis, femur, and arm. Our study indicated a positive correlation between serum 25‐hydroxyvitamin D and BMD in middle‐aged and older individuals. The impacts varied between different bone regions, with the trunk bone region having a greater impact than the radius region. These findings emphasize the importance of monitoring serum 25‐hydroxyvitamin D levels to optimize bone health.

## Graphical abstract

The study design, main research procedures, key findings, and clinical implications are briefly illustrated.

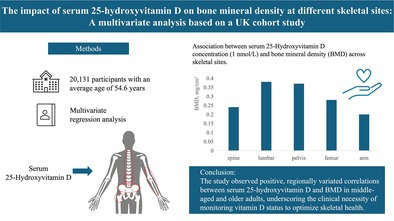

## 1 Introduction

Low bone mineral density (BMD) is the primary clinical manifestation and diagnostic variable of osteoporosis, a chronic disorder characterized by bone mass reduction and bone microarchitecture deterioration (Rachner et al. 2011). Age‐related bone loss is a prevalent phenomenon (Burr 2019; David et al. 2022), and the resulting fragile bone structure substantially elevates the susceptibility to fractures, thereby imposing a considerable disease burden on individuals and society (Wu et al. 2021).

Vitamin D is considered a vital element for maintaining bone health. Previous studies have demonstrated that sufficient vitamin D can inhibit the differentiation of osteoclast precursor cells into osteoclasts, effectively reducing bone resorption and conferring benefits to bone health (Christakos et al. 2019; Liang et al. 2022; Lips and van Schoor 2011). A population‐based bone dual‐energy X‐ray absorptiometry (DXA) screening study demonstrated that the peak value of BMD and the loss rate of BMD with age varied across different sites (Zeng et al. 2019), indicating that different sites of bone may have varied characteristics. High‐quality meta‐analyses of randomized clinical trials (RCT) found that vitamin D supplementation would help increase the BMD in the femur neck area but not at other sites (Reid et al. 2014). Furthermore, large‐scale trials, such as the VITAL trial, have demonstrated that supplemental vitamin D3 (2000 IU/day) did not result in significant bone density changes compared to placebo at the spine, hip, or total body in generally healthy adults (LeBoff et al. 2020). Consensus on the benefits of vitamin D supplementation for different sites or overall bone health remains elusive (Bischoff‐Ferrari et al. 2012; Kahwati et al. 2018; Zhao et al. 2017).

Several observational studies with cross‐sectional or case–control designs found serum vitamin D is associated with individual sites of BMD in the upper limb, femur, and spine (Bischoff‐Ferrari et al. 2004; Khodabakhshi et al. 2023; Liu et al. 2020; Øyen et al. 2011; Wang and Yang 2023). Meta‐analysis of RCTs investigating the impact of vitamin D supplementation on bone mineral density and fractures indicated that the benefits varied across bone sites (Bischoff‐Ferrari et al. 2012; Reid et al. 2014). Nevertheless, there are limited longitudinal studies on associations between serum vitamin D levels and bone densities. Few studies have examined these associations across various bone sites simultaneously. Functional and anatomical variations across the skeleton likely result in heterogeneous responses to vitamin D (Hart et al. 2020). Beyond differences in mechanical loading, skeletal sites vary significantly in microarchitecture and metabolic turnover rates. Furthermore, site‐specific variations in Vitamin D Receptor (VDR) expression and localized hormonal signaling may lead to divergent utilization of vitamin D across different regions (Bouillon et al. 2018). Therefore, simultaneously assessing multiple bone sites will provide a clearer distinction between systemic and site‐specific effects, a gap that remains unaddressed in current literature.

This study aims to evaluate the associations between serum vitamin D and bone mineral density at various anatomical sites and to examine whether a consistent relationship exists. The findings of this study could provide valuable insights that could significantly contribute to understanding and preventing bone mineral density loss.

## 2 Methods

### 2.1 Study Population and Data Source

Our study used data from the UK Biobank, a national prospective cohort. Detailed information about the cohort has been stated in previous studies (Sudlow et al. 2015; Thompson et al. 2025). Briefly introduced, this cohort enrolled approximately 500,000 participants in the United Kingdom aged 40 to 69 between 2006 and 2010. Consent was obtained from all participants for their involvement. A substantial amount of phenotypic information was gathered through questionnaires, physical evaluations, and biological samplings. Additionally, a variety of longitudinal health outcomes with data linking from multiple sources, including self‐reports, primary care records, hospital admissions, cancer register, and death record, were also offered. Data collection was executed by trained professionals using calibrated equipment and validated electronic questionnaires, adhering to strict UK Biobank protocols. Continuous quality assurance and validations were implemented throughout the recruitment and measurement phases to ensure the integrity and reproducibility (Fry et al. 2017; Sudlow et al. 2015; UK Biobank 2007).

UK Biobank performed dual‐energy X‐ray absorptiometry (DXA) examinations in a randomly selected subset of participants between 2014 and 2022 (Littlejohns et al. 2020). The present study included participants who underwent these DXA examinations. Participants' baseline characteristics, such as demographics, lifestyles, physical measurements, and lab tests, were collected from the UK Biobank datasets. Individuals who had no blood sample tests at the baseline (2006–2010), those who withdrew from the UK Biobank cohort and those who lacked complete data were excluded, with only complete cases were remained for the subsequent analysis. Baseline characteristics of the complete‐case cohort and excluded individuals were compared using standardized mean difference (SMD). An SMD of < 0.2 is considered indicative of a negligible difference between the groups (Table S1) (Austin 2009).

### 2.2 Assessment of Serum 25‐Hydroxyvitamin D Concentration

Serum 25‐hydroxyvitamin D is recognized as an effective metric for evaluating vitamin D status (Bischoff‐Ferrari et al. 2004; Feng et al. 2017). It was quantified in blood samples obtained at the time of participant recruitment between 2006 and 2010 using the DiaSorin Ltd. LIASON XL equipment, which employed a chemiluminescent immunoassay (CLIA) method. Quality control for the blood sample tests was overseen by UK Biobank (Resource 5636). The recorded serum 25‐hydroxyvitamin D values for each individual were extracted from data field 30890, with only reportable data (data field 30896) being included for the subsequent analysis.

### 2.3 Outcome and Covariates

Bone mineral density data were derived from DXA scans. In accordance with clinical guidelines for osteoporosis diagnosis and management, and informed by data availability, we assessed BMD at several key anatomical sites, including the whole spine, lumbar (L1–L4), pelvis, femur, and arm (Force 2018; UK Biobank 2015).

We took into account a variety of potential covariates, including socio‐demographic factors such as age, sex, ethnicity, highest level of education attained, annual household income before tax, and lifestyle factors including body mass index (BMI), smoking status, alcohol intake, physical activity levels, and index of multiple deprivation (IMD), which reflects a composite score of small area‐level socioeconomic indicators. Additionally, heelbone BMD at the baseline was also considered as a covariate. The estimated bone mineral density of the calcaneus was assessed via quantitative ultrasound using the Hologic Sahara Clinical Bone Sonometer, following a standardized protocol (UK Biobank 2011). The device automatically converts raw acoustic measures into a BMD value expressed in g/cm^2^.

### 2.4 Statistical Analysis

We presented continuous data as mean and standard deviation (SD) or median and interquartile range (IQR). Categorical variables were given as numbers and proportions. We used a multivariate mixed‐effects model with multiple outcomes to simultaneously assess the relationship between serum 25‐hydroxyvitamin D and BMD at different sites. A multivariate analysis is efficient for directly comparing the associations of an explanatory variable with multiple dependent variables (Ni et al. 2020; Snijders and Bosker 2011). This approach was employed to investigate the influence of vitamin D on bone mineral density (BMD) and to ascertain whether a similar effect size was observed at different bone sites.

Potential confounders were incrementally adjusted. Model 1 included basic demographic characteristics (age, sex [Male and Female], and ethnicity [White and Non‐White]) and baseline heel bone measurements. The latter served as a proxy for baseline skeletal status to mitigate residual confounding from unmeasured determinants of bone health. Model 2 additionally adjusted for individual‐level socioeconomic status, including annual household income before tax (< £18,000, £18,000 to £30,999, £31,000 to £51,999, £52,000 to £100,000, and £100,000 and above), and the highest educational qualification (University or college, A‐level, GSCE, and other). The main model, Model 3, additionally adjusted for area‐level socioeconomic status and lifestyle factors, including IMD (grouped in five quantiles), BMI categories (underweight [< 18.5 kg/m^2^], normal [18.5 to < 25 kg/m^2^], overweight [25 to < 30 kg/m^2^], and obese [30 kg/m^2^ or higher]), smoking status (previous smoker, current smoker, and never smoker), alcohol drinking frequency (daily or almost daily, moderate drinking, and never or special occasional), and physical activity level (light, medium, and heavy).

### 2.5 Stratification, Interaction and Sensitivity Analyses

To compare the serum vitamin D effects on BMD between males and females, we conducted a stratified analysis by sex. The modification effect of sex was assessed by adding the interaction term of sex and vitamin D level based on Model 3. In sensitivity analysis, we further adjusted for serum calcium levels to diminish the effects modified by calcium, if any.

All data analyses were performed with R software (version 4.3.1, R Foundation for Statistical Computing, Vienna, Austria). R package “nlme” was used for the multivariate analysis.

## 3 Results

**FIGURE 1:**
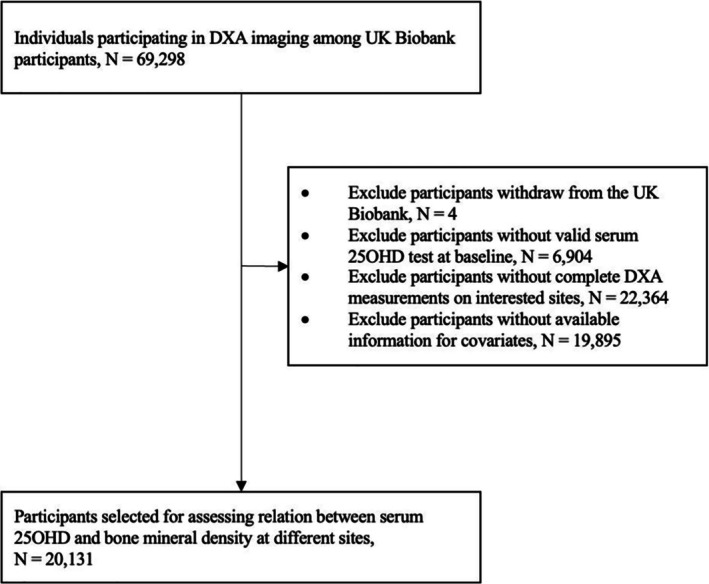
Flowchart of subject selection. 25OHD, 25‐hydroxyvitamin D.

**FIGURE 2:**
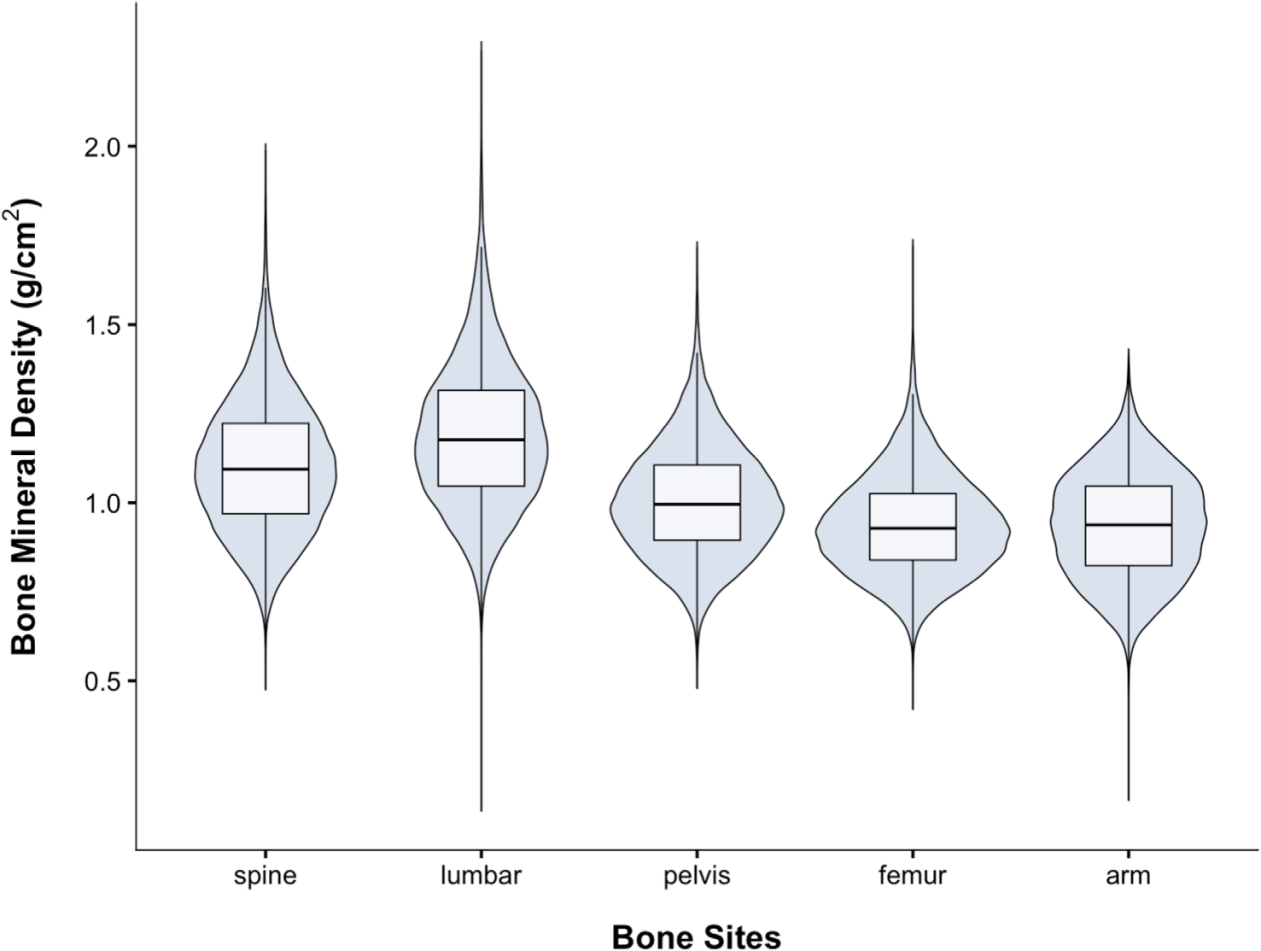
Bone mineral density at five sites (g/cm^2^).

The present study included a total of 20,131 participants (Figure 1). The study population was 48.9% female, with an average age [mean (SD)] of 54.56 (7.57) years old (Table 1). The mean serum 25‐hydroxyvitamin D concentration was 49.27 (20.64) nmol/L, which has been indicative of vitamin D deficiency (< 50 nmol/L) (Hossein‐nezhad and Holick 2013; Holick 2023). The majority of the participants were of White ethnicity (92.6%). It was observed that 37.0% of participants had an annual household income before tax of over £52,000, and 48.5% had obtained the highest university or college qualification. The prevalence of overweight and obesity was 60.2% among the participants. Over half of the participants never smoked (59.6%), 64.8% of participants consumed alcohol moderately, and 81% of individuals engaged in medium to high levels of physical activity. The mean bone mineral density (and SD) at the spine, lumbar, pelvis, femur and arm were 1.10 (0.18), 1.19 (0.20), 1.01 (0.15), 0.94 (0.14), and 0.94 (0.15) g/cm^2^, respectively (Figure 2).

**TABLE 1:** Characteristics of participants selected for analysis on the relationship between serum vitamin D and bone mineral density.

	Total: 20,131
Serum 25OHD vitamin D (nmol/L)	49.27 (20.64)
Heel bone mineral density (g/cm^2^)	0.56 (0.13)
Age (years), mean (SD)	54.56 (7.57)
Ethnicity	
White	18,648 (92.6)
Non‐white	1483 (7.4)
Sex	
Female	9834 (48.9)
Male	10,297 (51.1)
Household income, *N* (%)	
< £18,000	2233 (11.1)
£18,000 to £30,999	4367 (21.7)
£31,000 to £51,999	6086 (30.2)
£52,000 to 100,000	5820 (28.9)
> 100,000	1625 (8.1)
Education qualification, *N* (%)	
University or college	9759 (48.5)
A‐levels or equivalent	2667 (13.2)
GCSEs or equivalent	4540 (22.6)
Other	3165 (15.7)
Body mass index, *N* (%)	
Underweight (< 18.5 kg/m^2^)	91 (0.5)
Normal (18.5 to < 25 kg/m^2^)	7931 (39.4)
Overweight (25 to < 30 kg/m^2^)	8675 (43.1)
Obesity (≥ 30 kg/m^2^)	3434 (17.1)
Smoking status, *N* (%)	
Never	12,000 (59.6)
Previous	6784 (33.7)
Current	1347 (6.7)
Alcohol drinking, *N* (%)	
Daily or almost daily	2374 (11.8)
Moderate drinking	13,036 (64.8)
Never or special occasional	4721 (23.5)
Physical activity, *N* (%)	
Low	3835 (19.1)
Medium	8468 (42.1)
High	7828 (38.9)

The results of the multivariate regression analysis are presented in Table 2. In the main model (Model 3), adjusted for age, sex, ethnicity, heel bone BMD, household income level, education qualification, body mass index, smoke status, alcohol drink frequency, physical activity level, and index of multiple deprivation, we observed that an increase of 1 nmol/L in serum 25‐hydroxyvitamin D was associated with a 0.24, 0.38, 0.37, 0.28, and 0.20 mg/cm^2^ higher BMD at the spine, lumbar, pelvis, femur, and arm, respectively, when all other confounding variables were held constant. The multivariate regression demonstrated that the association between serum 25‐hydroxyvitamin D levels and bone mineral density exhibited regional heterogeneity. The associations between serum vitamin D exhibited a stronger correlation with the lumbar and pelvis, followed by the femur, spine, and arm.

**TABLE 2:** The associations between serum 25‐hydroxyvitamin D and different sites of bone mineral density.

Bone site	Coefficients (95% CI)	*p*
Model 1		
Spine	−0.11 (−0.21, −0.01)	0.032
Lumbar	0.09 (−0.03, 0.22)	0.123
Pelvis	0.20 (0.12, 0.29)	< 0.001
Femur	0.11 (0.03, 0.19)	0.008
Arm	0.06 (0.00, 0.12)	0.063
Model 2		
Spine	−0.16 (−0.26, −0.06)	0.001
Lumbar	0.05 (−0.07, 0.17)	0.384
Pelvis	0.18 (0.10, 0.27)	< 0.001
Femur	0.08 (0.00, 0.16)	0.046
Arm	0.02 (−0.04, 0.08)	0.550
Model 3		
Spine	0.24 (0.15, 0.34)	< 0.001
Lumbar	0.38 (0.26, 0.50)	< 0.001
Pelvis	0.37 (0.29, 0.45)	< 0.001
Femur	0.28 (0.20, 0.36)	< 0.001
Arm	0.20 (0.14, 0.26)	< 0.001

*Note:* Model 1: adjusted for age, sex, ethnicity and heel bone BMD. Model 2: Model 1 additionally adjusted for household income level, and education qualification. Model 3: Model 2 further adjusted for body mass index, smoke status, alcohol drink frequency, physical activity level, and index of multiple deprivation.

Abbreviations: BMD, bone mineral density; CI, confident interval.

**FIGURE 3:**
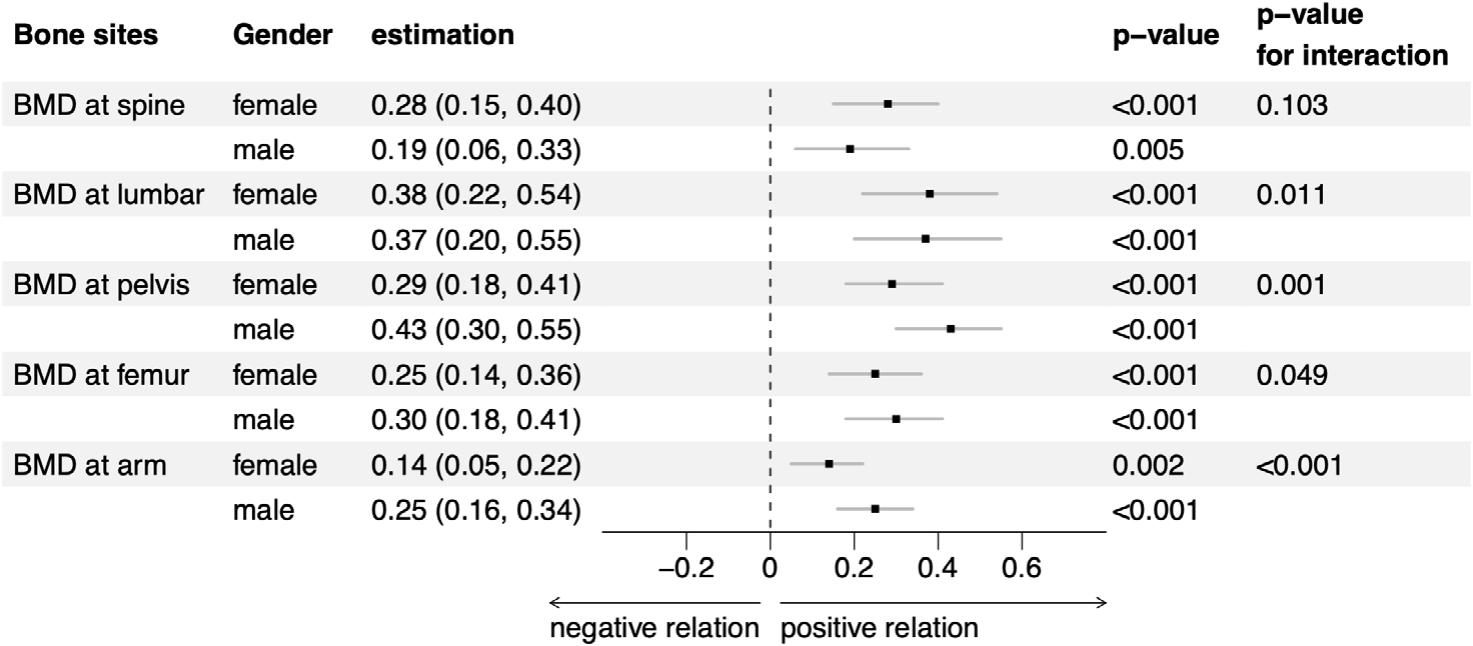
Subgroup analysis of the association between serum 25‐hydroxyvitamin D and five sites of bone mineral density by gender. BMD, bone mineral density.

A subgroup analysis by gender showed that there were differences in the association between 25‐hydroxyvitamin D and BMD by females and males (Figure 3). The associations between 25‐hydroxyvitamin D and arm BMD, pelvis BMD, and femur BMD differed by gender, with smaller effects observed in females compared to males (*p*‐value for interaction < 0.05). Specifically, the coefficients and 95% confidence intervals for females were 0.14 (0.05, 0.22), 0.29 (0.18, 0.41), and 0.25 (0.14, 0.36) for arm, pelvis, and femur BMD, respectively, while for males they were 0.25 (0.16, 0.34), 0.43 (0.30, 0.55), and 0.30 (0.18, 0.41). Conversely, the impact of serum 25‐hydroxyvitamin D on the lumbar system was slightly higher in females compared to males [0.38 (0.22, 0.54) vs. 0.37 (0.20, 0.55), *p*‐value for interaction = 0.011]. No significant gender differences were observed at the spine. The sensitivity analysis that further adjusted for serum calcium levels showed that the associations between vitamin D and BMD at multiple sites remained similar (Table 3). The correlations between vitamin D and vertebral bones were stronger than that of the arm bone.

**TABLE 3:** The sensitivity analysis of the associations between serum 25‐hydroxyvitamin D and different sites of bone mineral density (*N* = 18,438).

Bone site	Coefficients (95% CI)	*p*
Spine	0.23 (0.13, 0.32)	< 0.001
Lumbar	0.36 (0.24, 0.49)	< 0.001
Pelvis	0.36 (0.28, 0.45)	< 0.001
Femur	0.25 (0.16, 0.33)	< 0.001
Arm	0.19 (0.12, 0.25)	< 0.001

*Note:* The average concentration of serum calcium is 2.37 (standard deviation: 0.09) mmol/L. Model was adjusted for age, sex, heel bone BMD, ethnicity, household income level, education qualification, body mass index, smoke status, alcohol drink frequency, physical activity level and baseline serum calcium.

Abbreviations: BMD, bone mineral density; CI, confident interval.

## 4 Discussion

This retrospective cohort study observed 20,131 subjects over a median of 13.64 (IQR: 3.58) years. The results of the multivariate regression analysis revealed that baseline serum 25‐hydroxyvitamin D levels were positively associated with bone mineral density, demonstrating a stronger correlation with the lumbar and pelvis, followed by the femur, spine, and arm. Previous studies have investigated the link between vitamin D and bone mineral density at specific skeletal sites. Nevertheless, there is a paucity of research exploring the effects across multiple bone regions simultaneously. Our study is distinctive and innovative in that it addresses a notable research gap by simultaneously evaluating the associations between serum 25‐hydroxyvitamin D and multiple skeletal compartments in one multivariate model, allowing for a direct comparison of vitamin D's influence on various skeletal compartments within the same population. A more comprehensive and complete understanding of how multiple bone regions are affected by changes in vitamin D is obtained. Additionally, multiple testing on separate models can be avoided and type I error rate can be better controlled, compared to conducting a series of single analyses without accounting for multiple comparisons. Furthermore, serum 25‐hydroxyvitamin D levels provide a more accurate reflection of physiologically active concentrations than supplementation‐based data. This simultaneous assessment allows for a clearer distinction between systemic and region‐specific impacts, offering novel insights into the heterogeneous nature of vitamin D's role on bone health. Bone is subject to continuous turnover and remodeling throughout their lifespan. This process is distinguished by osteoblastic bone formation, which exerts an anabolic effect, and osteoclastic bone resorption, which exerts a catabolic effect. The 25‐hydroxyvitamin D plays a pivotal role in regulating bone formation and absorption (Goltzman 2018). Consequently, optimizing 25‐hydroxyvitamin D status would facilitate the normalization of bone turnover and enhance bone mineral density (Adami et al. 2009). Our findings are similar to those of earlier observational studies, which have demonstrated a positive association between serum vitamin D levels and single bone mineral density in the bone from the total hip, femur neck, distal radius, and upper limb (Bischoff‐Ferrari et al. 2004; Liu et al. 2020; Øyen et al. 2011). Our study specifically found serum 25‐hydroxyvitamin D positively associated with BMD at multiple bone sites and allowed the associations to be compared directly, including the hip bone area (lumbar, pelvis, and femur bone), the spine, and the upper limbs (arm bone), simultaneously.

This study observed different effects of vitamin D on variant skeletal areas. It is important to note that humans cannot synthesize vitamin D independently (National Institutes of Health 2023). Vitamin D should be activated into its active form and then transported to skeletal tissues through the bloodstream (DeLuca 2012). We hypothesized that anatomical discrepancies in functional roles may result in variations in the response of different skeletal tissues to vitamin D. For instance, the vertebral skeleton primarily bears body weight to provide support, while the hip skeleton is subjected to greater impact and force. Consequently, different anatomical regions exhibit inherent tendencies to benefit from vitamin D supplementation (Hart et al. 2020). Local hormones (e.g., parathyroid hormones and calcitonin) and VDR at variated bone sites may also influence the utilization of vitamin D, leading to varying responses to vitamin D (Bouillon et al. 2018). The subgroup analysis demonstrated that the effects of vitamin D on BMD differed between males and females. One potential explanation for this difference is attributed to the gender difference in vitamin D metabolism, which results in different serum levels and actions on bone health (Wierzbicka and Oczkowicz 2022). Furthermore, as the study subjects have a mean age of 54.46 years old, the hormone effects during menopause may also contribute to the finding that the association of vitamin D and BMD among females is smaller than that among males (Finkelstein et al. 2008; Park et al. 2021).

Our research identified positive correlations between serum vitamin D levels and BMD at various sites with different degrees, which is consistent with several previous RCT findings. One review on RCT of vitamin D supplementation and BMD demonstrated that femoral neck BMD experienced slight benefits, while no benefits were found at other bone sites (Reid et al. 2014). A pooled analysis of vitamin D and fracture prevention suggested that the hip or nonvertebral fractures would benefit from vitamin D supplementation when the dose was high (≥ 800 IU daily) (Bischoff‐Ferrari et al. 2012). However, another review showed that vitamin D supplementation would not decrease fracture risk among community‐dwelling older adults (Zhao et al. 2017). The inconsistent findings may be due to different baseline serum vitamin D before vitamin D supplementation. Administering additional vitamin D to individuals with adequate vitamin D levels is likely to produce no observable effect (Reid et al. 2014). Additionally, the relationship between vitamin D supplementation and the serum's active form of vitamin D is complex and often nonlinear (Bouillon et al. 2022; Heaney 2012). Vitamin D is synthesized through exposure to sunlight or dietary intake, with the two main forms being vitamin D_2_ and vitamin D_3_. However, both forms are inactive and require conversion to hydroxylated metabolites to exhibit hormonal activity through the liver and kidney (DeLuca 2012; Feldman et al. 2001). This process would be influenced by several factors, including baseline serum vitamin D levels, the absorption rate in the intestines, the responsiveness to the intermediate hormones, and the genetic effect of the vitamin D receptor (VDR) (Burt et al. 2019; Haussler et al. 2011; Silva and Furlanetto 2017). Any factor that varies during the process will result in a non‐linear impact of vitamin D supplementation and serum 25‐hydroxyvitamin D. In addition, bone mineral density could serve as an essential predictor of fracture risk, but the risk of fracture is not solely related to BMD. Other factors, such as bone strength, muscle functions, and neurofunction to balance, have also been identified as potential contributors to the protective effect of vitamin D in preventing fractures (Laird et al. 2010; Skuladottir et al. 2021).

Our study used the multivariate regression analysis and observed the positive association between serum 25‐hydroxyvitamin D and BMD. Concurrently, we directly compared the associations between vitamin D and BMD at multiple bone sites. The present study represents a novel approach to evaluating the effects of vitamin D on different skeletal areas, thereby providing a more comprehensive understanding of the prevention of bone density loss. Nevertheless, our study has several limitations. Firstly, UK Biobank was a cohort of voluntary participants, and it may have been subject to the “healthy volunteer” selection bias. This means that individuals of the cohort might have relatively better life habits, fewer self‐reported medical conditions, and higher socioeconomic levels than the general UK population. Nevertheless, the validation assessment revealed that the “healthy volunteer” bias of the UK Biobank may not influence the findings of “exposure‐disease” relationship analyses (Fry et al. 2017). Secondly, restricted by the data availability, we only used baseline serum 25‐hydroxyvitamin D level to represent vitamin status throughout the follow‐up period. While absolute 25‐hydroxyvitamin D levels can fluctuate, prior research indicates that an individual's relative “ranking” within a population remains moderately stable over long periods. This longitudinal consistency is evidenced by intraclass correlation coefficients of vitamin D ranging from 0.42 to 0.52 over 14 years (Jorde et al. 2010), with higher correlations of 0.60 to 0.73 reported over shorter intervals of 3 to 11 years (Mai et al. 2025; McKibben et al. 2016; Meng et al. 2012; Zhu et al. 2022). This stability is also supported in biological “set points” partially determined by stable genetic factors that persist despite behavioral shifts (Sutherland et al. 2022). Consequently, baseline concentrations permit reliable discrimination of individuals with distinct long‐term vitamin D status and serve as a reasonable proxy for assessing its long‐term impact on bone mineral density. Thirdly, despite adjusting for key covariates in the full model, potential confounding form unobserved factors may still exist. Lastly, most participants in the UK Biobank are white people, and the generalizability of our results to other ethnic populations needs to be validated.

## 5 Conclusions

With a prospective cohort, our study contributed valuable evidence supporting positive correlations between serum 25‐hydroxyvitamin D levels and bone mineral density at multiple sites in the middle‐aged and elderly population. The observed variations in the associations between serum 25‐hydroxyvitamin D levels and bone mineral density across different bone regions showed a stronger association at the trunk bone areas compared to the radial bones. Additionally, it indicated that females and males may experience different benefits from serum 25‐hydroxyvitamin D with respect to bone health. Notably, as the study population was predominantly White (92.6%), the above findings may not be directly generalizable to other ethnic groups. Further research conducted in different ethnicities is warranted. These findings highlight the importance of serum 25‐hydroxyvitamin D measurement in order to maintain bone health.

## Author Contributions

**Shuangnan Cui:** writing – original draft, formal analysis, visualization, data curation, validation. **Xiaoxin I. Yao:** writing – review and editing, project administration, conceptualization, methodology, funding acquisition, writing – original draft, supervision, data curation, resources. **Hualian Zeng:** writing – original draft, formal analysis, visualization, data curation, validation. **Qisheng Lin:** project administration, funding acquisition. **Chen Shen:** writing – review and editing. **Xinning Tong:** visualization, investigation, funding acquisition, writing – original draft, formal analysis, validation. **Yuejiao Huang:** project administration, funding acquisition. **Jinhui Li:** writing – review and editing.

## Funding

This work was supported by Futian Healthcare Research Project, China (grant number FTWS2025030 and FTWS2021014), Shenzhen Science and Technology Program, China (grant number JCYJ20220530144403007 and JCYJ20230807111016033); Guangdong Basic and Applied Basic Research Foundation, China (grant number 2023A1515011555 and 2023A1515110720); the National Natural Science Foundation of China (grant number 82103909); and Guangdong Provincial Clinical Research Center for Orthopedic Diseases (grant number 2023B110001). The funding sources played no role in the study design, in the collection, analysis, and interpretation of data, in the writing of the report, and neither in the decision to submit the paper for publication.

## Ethics Statement

UK Biobank was approved by the North West Multi‐Centre Research Ethics Committee (MREC) (Reference 16/NW/0274).

## Consent

All participants in this study provided written informed consent.

## Conflicts of Interest

The authors declare no conflicts of interest.

## Supporting information


**Table S1:** Characteristics of participants included and excluded from the analysis.

## Data Availability

The data that support the findings of this study are available from UK Biobank. Restrictions apply to the availability of these data, which were used under license for this study. Data are available from https://www.ukbiobank.ac.uk/enable‐your‐research/apply‐for‐access with the permission of UK Biobank.
