# Supplementary material for: The Impact of Serum 25‐Hydroxyvitamin D on Bone Mineral Density at Different Skeletal Sites: A Multivariate Analysis Based on a UK Cohort Study
Source: Food Sci Nutr. 2026 May 17;14(5):e71894. doi: 10.1002/fsn3.71894 (PMC13180784; doi:10.1002/fsn3.71894)
Supplement: Supplementary file 1 — Table S1: Characteristics of participants included and excluded from the analysis. [file FSN3-14-e71894-s001.docx]

Supplementary Table 1. Characteristics of participants included and excluded from the analysis

|  | Included participants | Excluded participants | SMD |
| --- | --- | --- | --- |
|  | 20131 | 49163 |  |
| **Serum 25OHD vitamin D** (nmol/L) | 49.27 (20.64) | 50.29 (20.95) | 0.049 |
| **Heel bone mineral density** (g/cm^2^) | 0.56 (0.13) | 0.55 (0.13) | 0.041 |
| **Age** (years), Mean (SD) | 54.56 (7.57) | 54.73 (7.52) | 0.023 |
| **Ethnicity** |  |  | 0.026 |
| White | 18648 (92.6) | 45001 (91.9) |  |
| Non-white | 1483 (7.4) | 4612 (8.1) |  |
| **Sex** |  |  | 0.084 |
| Female | 9834 (48.9) | 26086 (53.1) |  |
| Male | 10297 (51.1) | 23077 (46.9) |  |
| **Household income N (%)** |  |  | 0.019 |
| Less than £18,000 | 2233 (11.1) | 4914 (11.6) |  |
| £18,000 to £30,999 | 4367 (21.7) | 8980 (21.2) |  |
| £31,000 to £51,999 | 6086 (30.2) | 12690 (30.0) |  |
| £52,000 to 100,000 | 5820 (28.9) | 12294 (29.1) |  |
| Greater than 100,000 | 1625 (8.1) | 3436 (8.1) |  |
| **Education qualification N (%)** |  |  | 0.096 |
| University or college | 9759 (48.5) | 21207 (44.1) |  |
| A-levels or equivalent | 2667 (13.2) | 6273 (13.1) |  |
| GCSEs or equivalent | 4540 (22.6) | 12009 (25.0) |  |
| Other | 3165 (15.7) | 8567 (17.8) |  |
| **Body mass index** **N (%)** |  |  | 0.042 |
| Underweight (<18.5 kg/m^2^) | 91 (0.5) | 232 (0.5) |  |
| Normal (18.5 to <25 kg/m^2^) | 7931 (39.4) | 18885 (38.5) |  |
| Overweight (25 to <30 kg/m^2^) | 8675 (43.1) | 20783 (42.4) |  |
| Obesity (≥30 kg/m^2^) | 3434 (17.1) | 9149 (18.7) |  |
| **Smoking status N (%)** |  |  | 0.018 |
| Never | 12000 (59.6) | 29634 (60.5) |  |
| Previous | 6784 (33.7) | 16117 (32.9) |  |
| Current | 1347 (6.7) | 3239 (6.6) |  |
| **Alcohol drinking N (%)** |  |  | 0.054 |
| Daily or almost daily | 2374 (11.8) | 6593 (13.4) |  |
| Moderate drinking | 13036 (64.8) | 31667 (64.5) |  |
| Never or special occasional | 4721 (23.5) | 10862 (22.1) |  |
| **Physical activity N (%)** |  |  | 0.033 |
| Low | 3835 (19.1) | 6984 (18.0) |  |
| Medium | 8468 (42.1) | 16222 (41.8) |  |
| High | 7828 (38.9) | 15639 (40.3) |  |

SMD: standardized mean difference
